# British Society of Breast Radiology Virtual Annual Scientific Meeting 2021

**DOI:** 10.1186/s13058-021-01471-2

**Published:** 2021-11-01

## Research Work in Progress

### Novel software tools for extracting additional clinical value from DBT reconstructions

#### Simon Lowes^1,2^, David Joyce^3^, Ben Lopez^3^, Paul Scott^3^, Alice Leaver^1^

##### ^1^Gateshead Health NHS Foundation Trust, Gateshead, United Kingdom. ^2^Newcastle University, Newcastle upon Tyne, United Kingdom. ^3^IBEX Innovations Ltd, Sedgefield, United Kingdom

###### Correspondence: David Joyce

*Breast Cancer Research* 23(2)

X-Ray scatter is a well-known problem in mammography and DBT, complicating image presentation and impacting lesion contrast. Physical anti-scatter grids are commonly used to minimise the negative effects, but with the penalty of increased radiation dose. However, rather than being a problem to be eliminated, the scatter signal can be used advantageously to provide an information channel which can be harnessed to extract additional clinical value from DBT reconstructions.

A physics model of the mammography system can be used to build, in real time, a composition and thickness map of the breast. This information can be used to correct for X-ray scatter without the need for a grid, resulting in reduced radiation dose and simplified system hardware. SDNR measurements following the EUREF guideline method indicate grid equivalence at a 25% dose reduction for breast equivalent thicknesses up to 90mm. In DBT, where it is less common for vendors to manage X-ray scatter, the quality of synthetic 2D images can also be improved, particularly for thicker breasts.

The model also facilitates reconstruction of 3D images with near isotropic resolution. Avoiding depth artefacts typical in standard DBT reconstruction methods may help in discriminating between features in dense breasts; the composition information may facilitate differentiation between glandular tissue, cysts, and other materials in the breast including contrast agent.

We will provide an overview of the methodology and some early results demonstrating the potential of these software tools. We will also present a vision for some of the emerging opportunities arising from these techniques.

## Scientific—Axilla

### New pulse biopsy device proved to be safe and effective in axillary lymph nodes with pulsed insertion facilitating enhanced needle control: Initial results from the German multi-center PULSE study

#### Marc Thill^1^, Thorsten Kühn^2^, Uwe Peisker^3^, Angrit Stachs^4^, Mattea Reinisch^5^, Wolfram Malter^6^, Ines Gruber^7^, Stefan Paepke^8^, Magnus Olsen^9^, Kai-Uwe Schässburger^9^

##### ^1^Agaplesion Markus Hospital, Frankfurt, Germany. ^2^Klinikum Esslingen, Esslingen, Germany. ^3^Hermann-Josef-Krankenhaus, Erkelenz, Germany. ^4^Klinikum Südstadt Rostock, Rostock, Germany. ^5^Klinken Essen Mitte, Essen, Germany. ^6^University Hospital Köln, Köln, Germany. ^7^University Hospital Tübingen, Tübingen, Germany. ^8^University Hospital r.d. Isar of TU München, München, Germany. ^9^Neodynamics, Lidingö, Sweden

###### Correspondence: Marc Thill

*Breast Cancer Research* 23(2)

**Purpose** Core needle biopsy (CNB) and vacuum-assisted biopsy (VAB) have inherent design drawbacks regarding needle control, tissue yield and unnecessary tissue trauma. The purpose of this study is to document the performance of a newly developed 14G vacuum-assisted open-tip biopsy device intended for increased tissue yield and controlled needle insertion in the axillary lymph nodes.

**Materials and Methods** For this ethically approved German prospective multi-center study, patients with clinically/sonographically suspicious axillary lymph nodes at the time of breast cancer diagnosis underwent minimally invasive lymph node tissue sampling following written informed consent. The biopsy device (NeoNavia Biopsy System, NeoDynamics AB, Sweden) incorporates pneumatic pulse technology to provide controlled needle insertion and accurate lesion targeting. Primary outcome was success rate (tissue sampled from the lymph node). Complications, detailed procedure data and user experience were recorded. Analysis is based on biopsy data from 115 patients.

**Results** Mean age of the cohort was 57.2 years with a mean lymph node size of 18.4 mm. Success rate for tissue sampling from the lymph node was 92% (106/115). Hematoma in the axilla occurred in 1.7% (2/115) of patients (one mild, one moderate). Pain in the axilla was reported in 2.6% (3/115) of patients (two mild, one moderate).

**Conclusions** The biopsy device was safe and effective for use in the axillary lymph nodes in a broad patient cohort. Pulses were perceived to stabilize the targeted lymph node and enhance needle control during insertion. It was possible to obtain multiple samples with a single insertion.

## Scientific—Breast MR

### Investigation of Sternal Lesions Identified by Breast MRI

#### Fotini Asprou, Hassan Rehman, Zatinahhayu Mohd Isa

##### Royal Stoke University Hospital, University Hospital of North Midlands NHS Trust, Stoke-On-Trent, United Kingdom

###### Correspondence: Fotini Asprou

*Breast Cancer Research* 23(2)

The sternum is a frequent site for breast MRI identified bony lesions. These can result in additional imaging, change treatment intent, delay management, and increase patient anxiety. A small number of studies have suggested MRI characteristics more likely in metastatic lesions. We aim to compare the breast MRI characteristics of sternal lesions in those deemed benign versus malignant on investigation.

All breast MRI reports conducted in a single University Hospital over 2 years were retrospectively reviewed, with 16 sternal lesions identified in 756 breast MRIs.

11 underwent further CT, 3 sternal MRI, 1 PET/CT, and in 1 prior CT was used for characterisation. 13 (81%) were deemed benign and 3 (19%) malignant. All malignant lesions had further bony lesions. All 3 malignant lesions displayed high T2/STIR signal and 2 showed contrast enhancement (in 1 no post contrast imaging was available). 10 benign lesions displayed highT2/STIR signal with contrast enhancement, 1 high T2/STIR but no post contrast imaging available, 1 low T2/STIR with contrast enhancement, and in 1 contrast enhancement only. Type 1 and 2 enhancement curves were seen in 7 (58%) and 1 (8%) respectively of benign lesions, versus 0 malignant lesions. Type 3 enhancement curves were seen in 4 (33%) benign, versus 2 (100%) malignant lesions.

Metastatic sternal lesions are frequently identified in context with other skeletal lesions. High T2/STIR signal and contrast enhancement are common in both benign and malignant lesions. However, Type 3 dynamic enhancement curves, whilst not specific for malignancy, should heighten suspicion for metastasis.

## Scientific—Breast US

### Management of additional lesions detected on Staging and Problem-solving Breast MRI – DGH Experience

#### Tania Mercado, Nithya Vidyaprakash, Asha Eleti

##### Southend University Hospital, Southend on Sea, United Kingdom

###### Correspondence: Tania Mercado

*Breast Cancer Research* 23(2)

Contrast-enhanced breast MRI possesses high sensitivity for diagnosing breast cancer and identifying additional lesions not detected on previous imaging studies. Nevertheless, due to its lower specificity further evaluation of these lesions is required. Second look ultrasound is a convenient and low-cost imaging technique. This work aims to study the correlation between second-look ultrasonography and preoperative / problem-solving breast MRIs and their pathology results. A retrospective study between January 2019 and December 2020 revealed that 205 Breast MRIs were performed for these purposes. 69 additional findings were reported in 54 MRIs. The most common findings were additional masses (52) and non-mass enhancement (13). Most of the lesions were classified MRI 3 (43) and MRI 4 (19) and the majority (34) measured 5-10 mm. Regarding the location, 24 lesions were in a different quadrant than the index lesion and 23 were located in the contralateral breast.

Second look US was performed in 47 patients with 59 lesions, having 88% of concordance with MRI findings. 17 lesions were classified as U2, therefore biopsies were not performed. US-guided biopsy was performed in 34 lesions revealing malignancy in 18. Seven lesions were not visible on ultrasound and were investigated using stereotactic/MRI guided biopsies and resulted in B2 lesions. MRI follow up in 6 months was indicated in two cases demonstrating stability. Findings suggest that second look ultrasound remains to be an important tool to assess MRI additional findings.

### Audit on the efficacy and accuracy of the elastography usage in conjunction with B- mode ultrasound for suspected breast lumps in the symptomatic one stop breast clinic setting

#### Guneesh Dadayal, Sophie Levine, Susan Oates, Rachael Brass

##### Airedale General Hospital, Keighley, United Kingdom

###### Correspondence: Guneesh Dadayal

*Breast Cancer Research* 23(2)

**Aim** The aim of the study was to evaluate the effectiveness and accuracy of the elastography usage in detecting suspected benign and malignant breast lesions in the symptomatic one stop breast clinics. The aim was also to evaluate the change of ultrasound’s BIRADS classification following elastography application in these patients.

**Methodology** The study was performed prospectively over a period of one year between May 2019 till May 2020 in the breast unit of the radiology department of the district general hospital. Total 133 consecutive women who underwent ultrasound guided breast biopsy during this period were found and included in the study with an age range of 18–90 years. Any patient who could not have a biopsy for a suspected breast lump was excluded. Variables like resistive index (soft/ mixed/ hard) and size (equal/ smaller / larger) were used for elastography evaluation with B—mode ultrasound.

**Results** Amongst 133 women, 93 were over 47 years and 32 were between 31–47 years. Out of total 133 women with suspected breast lump, 58 women (44%) had histological proven benign lump, mostly fibroadenoma. The sensitivity of elastography in detecting suspected benign pathology was 78% (45/58). The remaining 75 women had biopsy proven malignant lump. The sensitivity of the elastography in detecting suspected malignant pathology was 87% (65/75).

**Conclusion** Elastography has a better sensitivity in detecting malignant (87%) pathology than benign (78%) especially when both of its variables i.e. resistive index and size criteria are applied with B-mode ultrasound scanning.

### Single centre assessment of usefulness of shear- wave elastography in assessment of symptomatic breast lesions

#### Ajit Dhillon, Eddie Gibson, Calvin Ng

##### Antrim Area Hospital, Antrim, United Kingdom

###### Correspondence: Ajit Dhillon

*Breast Cancer Research* 23(2).

**Aim** To determine whether the addition of shear wave elastography (SWE) to standard B- mode ultrasound assessment of symptomatic breast lesions can influence selection of patients undergoing fine needle aspiration (FNA) or core biopsy.

**Methods** Retrospective review of data of patients who underwent SWE assessment carried out by 2 consultant breast radiologists. Patient demographics, lesion size, BI- RADS classification, FNA or core biopsy carried out, SWE evaluation with Emean and standard deviation (SD), and pathology.

**Results** A total of 152 women with 162 masses were assessed. Median age of the cohort was 57 years (range 15- 62 years). Overall, the average SWE Emean 43.68 (range 0- 108.60) with median SD 2.45 (range 0–18.40). In total 90 patients underwent FNA or core biopsy. Median lesion size was 16 mm (range 4- 55 mm). BIRADS 2 in 123 patients (80.9%) with median 16 mm (range 4- 41 mm), average SWE Emean 22.36 (range 1.73- 62.40) and SD 2.46 (range 0- 18.4) with malignancy confirmed in 4 patients. BIRADS 3 in 28 patients (18.42%) with median lesion size 16 mm (range 8- 50 mm), average SWE Emean 22.36 (range 0- 108.60) and SD 2.46 (range 0- 14.60) with malignancy in 2 patients. Of the cohort, 6 patients (3.9%) had malignancy confirmed on pathology with median age 39.5 years (range 31- 56 years). Median lesion size 13 mm (range 6- 22 mm), average SWE Emean 25.63 (range 1.73- 78.13) and SD 1.51 (range 1.15- 13.50).

**Conclusion** Incorporation of SWE findings did not aid patient selection for or against FNA or core biopsy.

## Scientific—High Risk Screening

### Very High-risk breast screening at King’s College Hospital - A 5-year review

#### Monica Patil, Clare Peacock, Shalini Wijesuriya, Michael Michell, Rema Wasan, Keshtra Satchithananda, Nikhil Patel, Bhavna Batohi, Rumana Rahim, Juliet Morel

##### King's College Hospital, London, United Kingdom

###### Correspondence: Monica Patil

*Breast Cancer Research* 23(2).

**Aim** Our aim was to review the Very High-risk (VHR) breast screening programme at King’s College Hospital, London from 2015 to 2020 and study the cancer detection rates (CDR), outcomes and modalities of detection.

**Methodology** Data was collected retrospectively from NHS BSP and PACS. All the cancers detected through the VHR screening from 2015 to 2020 were included. Protocols for imaging were as per NHS BSP VHR guidelines.

**Observations** 1052 women attended the VHR screening programme, majority were BRCA1 (476) and BRCA2 (437). Others were tp53 (9), supradiaphragmatic radiation (85; SRT) and high-risk equivalents (45). We detected 19 malignancies (7 BRCA1, 8 BRCA2, 1 BRCA1&2, 1 SRT, 1 VHR equivalent, 1 tp53). All BRCA1 patients had soft tissue masses which were Grade 3 invasive ductal carcinoma (IDC). These cancers were all less than 2 cm in size. 3 of these patients were less than 35-year-old. 5 BRCA2 patients had IDC, 2 had high grade ductal carcinoma in situ (DCIS) and 1 had metastasis from an ovarian cancer. Other very high-risk groups had IDC. Ultrasound visible target for biopsy was seen in 17 patients. 1 patient had microcalcifications, needing tomosynthesis guided biopsy. 1 patient had non mass enhancement, occult on ultrasound and mammograms, needing MRI guided biopsy. All patients had a normal axillary ultrasound. CDR (per 1000) was 15 in BRCA1 and 18 in BRCA2.

**Conclusion** All BRCA1 cancers were grade 3 IDC soft tissue masses. All the primary malignancies were IDC. Cancer in less than 35-year-olds was detected only in BRCA1.

## Scientific—Mammography

### The creation of a breast screening image database – The Cambridge Cohort – Mammography East-Anglia Digital Imaging Archive (CC-MEDIA)

#### Sarah Hickman^1^, Sue Hudson^2^, Nicholas Payne^1^, Richard Black^3^, Bahman Kasmai^4^, Andrew Priest^3^, Arne Juette^4^, Fiona Gilbert^1,3^

##### ^1^University of Cambridge, Cambridge, United Kingdom. ^2^PAS Consulting, London, United Kingdom. ^3^Cambridge University Hospitals NHS Foundation Trust, Cambridge, United Kingdom. ^4^Norfolk and Norwich University Hospitals NHS Foundation Trust, Norwich, United Kingdom

###### Correspondence: Sarah Hickman

*Breast Cancer Research* 23(2).

**Background** Medical imaging databases are increasingly important for artificial intelligence algorithm development and testing. External datasets that provide an unseen, representative cohort of breast screening for benchmark testing are sparsely available.

**Methods** We are building a medical imaging database consisting of breast screening data collected at two NHS Breast Screening Programme sites between 2011 and 2022. This data includes the standard two-view mammogram screening images as well as additional views and raw data where available. Clinical outcomes in our database are representative of the screening population, with a ground truth provided through clinical metadata follow up and / or histopathological results. Multiple oversight steps have been taken to create this ‘Controlled Database’ including; ethical approval, a Database Access Committee to supervise information governance (IG), and patient and public involvement to design patient facing material. Clinical metadata is accessed using specialised queries for the National Breast Screening System (NBSS). Picture Archiving Communication Systems (PACS) are queried for the mammographic images to be retrieved and de-identified in Digital Imaging and Communications in Medicine (DICOM) format. All clinical metadata and DICOM images are then securely stored and processed at the University of Cambridge.

**Results or Findings** The de-identified database to date contains 20,863 exams including normal and benign cases as well as interval, screen-detected and subsequent-round cancers.

**Conclusion** Creating a medical imaging database is a complex task requiring expertise from IT and IG. Our database provides an independent research environment for benchmark testing to compare and provide feedback for different artificial intelligence algorithms.

### Analysis of enhancement intensity and patterns of benign and malignant lesions on contrast-enhanced Spectral Mammography

#### Anam Ali, Tamara Suaris

##### Barts Health NHS Trust, London, United Kingdom

###### Correspondence: Anam Ali

*Breast Cancer Research* 23(2).

**Introduction** Contrast-enhanced spectral mammography (CESM) is a new method for assessment of breast cancer.

**Aim** The aim of this study was to evaluate the performance of CESM compared with digital MG and breast magnetic resonance imaging (MRI) in the pre-operative assessment of women with breast cancer.

**Methods** A retrospective analysis of patients undergoing CESM at Barts Health NHS trust between November 2020 and June 2021 was performed. CESM was matched with MRI for all patients and both occurred within days of each other. All patients had suspicious lesions found on either MG and breast ultrasound. All lesions with correlating histopathological proven diagnoses were included in the study. The degree of contrast enhancement on CESM was quantitatively assessed by measuring the region of interest (ROI) difference between the enhancing lesion and background as a ratio on both craniocaudal (CC) and mediolateral (ML) projections.

**Results** A total of 28 female patients underwent CESM and from these, 40 lesions were detected. Amongst these, 22 (55%) were assessed to be invasive cancers, 14 (35%) were non-invasive cancers, and 4 (25%) were benign. Analysis of enhancement indices showed the following mean ROI signal ratios: invasive cancers (1.60); non-invasive (1.28); benign (1.07).

**Conclusions** This study has demonstrated a correlation between the degree of lesion enhancement in CESM and malignant and benign lesions. Invasive malignant lesions had a stronger degree enhancement than benign lesions. Quantitative analysis of enhancement levels in CESM is a feasible practice comparable to MRI in the pre-operative assessment of women with breast cancer.

### Audit of DBT guided biopsies in ultrasound occult soft tissue abnormalities - A 3-year review

#### Monica Patil, Rema Wasan, Michael Michell, Shalini Wijesuriya, Clare Peacock, Nikhil Patel, Rumana Rahim, Bhavna Batohi, Keshtra Satchithananda, Juliet Morel

##### King's College Hospital, London, United Kingdom

###### Correspondence: Monica Patil

*Breast Cancer Research* 23(2).

**Introduction** A small proportion of mammographically detected soft tissue abnormalities are occult on ultrasound. In this study, we correlate imaging findings of these lesions with pathology outcome over a 3-year period.

**Method** Consecutive DBT guided vacuum assisted biopsies performed for ultrasound occult soft tissue lesions from January 2018 to December 2020 were included. Mammographic suspicion (M1 to M5), mammographic sign, lesion size and pathology were recorded. Two experienced breast screening radiologists, blinded to pathology, read the 2D digital mammograms and DBT images. The assessment DBT provided the final score.

**Results** 40/1334 (3%) DBT guided biopsies were performed for ultrasound occult soft tissue lesions. 24/40 (60%) were distortions, 10/40 (25%) were masses and 6/40 (15%) were asymmetric densities (ASD). Of the 24 distortions, 1 was graded M5 (CSL), 18 were M4 (1 invasive cancer, 1 DCIS, 1 LCIS, 15 benign), 5 were M3 (all benign). Of the 10 masses, 1 was graded M5 (malignant), 2 were graded M4 (1 malignant, 1 papilloma), 7 were graded M3 (all benign). Of the 6 ASD, 2 were graded M4 (1 invasive lobular carcinoma, 1 LCIS). The remaining 4 were graded M3 (all benign). Single view abnormalities (5 patients) had a benign histology. All M3 abnormalities were benign. All four cancers were graded as M4 or M5.

**Conclusion** In our cohort, 10% of ultrasound occult soft tissue lesions were malignant, emphasising the value of DBT guided biopsy in obtaining the diagnosis. M3 suspicion score and single view DBT abnormalities correlated with a benign outcome.

## Scientific—Procedures

### A single centre experience of providing an MRI guided breast biopsy service

#### Dr Maame Akua Akobeng, Dr Trupti Kulkarni, Dr Caroline Parkin

##### The Nightingale Centre, Wythenshawe Hospital, Manchester, United Kingdom

###### Correspondence: Dr Maame Akua Akobeng

*Breast Cancer Research* 23(2).

**Background** Contrast enhanced breast MRI is exceptionally sensitive. It can identify breast abnormalities that may not be discernible on other modalities. In such cases, MRI guided biopsy (MRIBX) has scope to identify foci of malignant breast disease that might only be seen on MRI. Evidence on the value of breast MRI is abundant. Data on the efficacy and impact of MRIBX services is less common. These results depict our MRIBX experience as a large breast unit serving our local population and referrals from across the country.

**Methods** A retrospective review of hospital records was performed to identify patients who had been appointed to undergo MRIBX. Patient records were reviewed to ascertain patient demographics, clinical details, target lesion characteristics, technical approach used, complications and ultimate patient outcomes.

**Results** Between Dec 2014 and July 2021, 216 appointments were attended for an MRI guided breast biopsy at our unit. In 169 instances the lesion to be targeted was reproduced, technically accessible and MRIBX was performed. Typically 18 samples were retrieved using a 10G EnCor VAB needle. Of the 169 MRIBXs performed, 43 yielded a malignant result. 3 patients developed haematomas.

**Conclusion** MRI guided breast biopsy is a safe and effective technique. It facilitates the diagnosis of subtle breast carcinomas and assists with surgical planning. Whilst sharing our experience we reflect in greater detail on our data, the highlights and challenges associated with MRIBX and its impact on patient outcomes.

### Localizing non-palpable breast lesions in a pandemic: RFID Tag evaluation

#### Ivy Okereke, Rupika Mehta, Asma Javed, Anil Madhavan, Chidinma Igu, Jocelyn Jaudalso, Rachel Merrett

##### Medway Maritime Hospital, Gillingham, Kent, United Kingdom

###### Correspondence: Ivy Okereke

*Breast Cancer Research* 23(2).

**Introduction** Currently in many breast units, wire guided localisation (WGL) is commonly used for localisation of non-palpable lesions. Recently, there has been a shift from tumour guided wire to non-wire localisation. This move could not have come at a better time than during COVID pandemic with all its attendant challenges. The aim of this study is to evaluate RFID tags as an effective alternative to wire guided localisation.

**Methods** This technique was evaluated in a prospective cohort of 20 patients needing localisation of non-palpable lesion. The lesion varied from masses, distortions to calcifications. The exclusion criteria were deep lesions, due to the tag reader limitations. The focus of the evaluation was: successful deployment and retrieval of tag, status of surgical margins, tag migration rate, acceptance of new procedure by radiologists and surgeons and patient’s compliance.

**Results** 18 tags were successfully inserted. Tag could not be deployed in two patients due to tumour size and consistency and lesion fragmentation following neo-adjuvant chemotherapy. Tag retrieval was performed by three surgeons. 5 out of the 18 patients had surgery at a different site due to capacity limitations at the height of the pandemic. Out of the 18 patients, only one needed re-excision for clear margins.

**Conclusion** Our study demonstrates that RFID is an effective and time-efficient alternative to WGL, with low margin positivity and re-operation rates, and high acceptance from all involved professional groups. RFID is useful for multi-site operation which was the case at the hospital during the COVID pandemic.

### Localisation of non-palpable breast lesions prior to surgical excision: An evaluation of ultrasound guided RFID tag localisation vs guidewire localisation

#### Louise Dodgeon

##### North Cumbria Integrated Care NHS Foundation Trust, Carlisle, United Kingdom

###### Correspondence: Louise Dodgeon

*Breast Cancer Research* 23(2).

**Introduction** Radiofrequency Identification (RFID) tag localisation is a new method of localising non-palpable breast lesions prior to surgery.The RFID tag is a miniature radiofrequency device which can be inserted into the breast at any time, as opposed to guidewire localisation which must be performed on the day of surgery. 

**Aim** NCIC NHS Foundation Trust began using RFID tags in December 2019; we sought to evaluate whether RFID tag localisation is comparable to wire localisation.

**Methods** A retrospective study compared the first 50 ultrasound guided RFID Tag localisations to the previous 50 ultrasound guidewire localisations. Accurate placement of both was evaluated, alongside involved post-operative surgical margins and re-excision rates.

**Results** All RFID tags and localisation wires were successfully deployed. Mean distance from target lesion was 0.68 mm for RFID tags vs 0.82 mm for guidewires. Presence of involved margins on resected surgical specimens was 4% (2/50) for RFID tags vs 12% (6/50) for guidewires (p = 0.140). Re-excision rates were 4% (2/50) for RFID tags vs 10% (5/50) for guidewires (p = 0.239).

**Conclusion** Although a lower incidence of involved margins and re-excision rates is seen with RFID tags compared to guidewires, this is not statistically significant due to the small sample size. However, the use of RFID tags allowed continuation of breast cancer surgery during the Coronavirus pandemic, which would not have been possible with guidewires which must be placed on the day of surgery. RFID tags are a preferable alternative to guidewires, benefiting the patient as well as radiology and surgical teams.

### Optimal needle assessment for M3-M5 microcalcification

#### Ivy Okereke

##### MEDWAY MARITIME HOSPITAL, GILLINGHAM, United Kingdom

###### Correspondence: Ivy Okereke

*Breast Cancer Research* 23(2).

**Introduction** Core needle biopsy (CNB) is the method of choice for sampling of M3-M5 microcalcifications (MCCs.) The two commonly used methods for obtaining pre-operative diagnosis of MCCs are stereotactic 14G CNB and 10G/11G Vacuum-assisted biopsy (VAB). There are different views as to which CNB type is optimal for accurately diagnosing MCCs. 

**Aim** The aim of the study is to establish if there is a relationship between CNB type and the accurate diagnosis of MCC.

**Methods** A retrospective review was undertaken of all clients who were recalled for assessment of screen-detected MCC with an M3-M5 grading and who underwent a stereotactic core biopsy (SCB) with either 14G needle or 10G VAB needle between 2016 and 2018. Exclusions included clients who had multifocal MCCs and those who had M3-M5 MCCs with associated mammographic or ultrasound abnormality. 221 cases of SCB were used for this study with a distribution of 112 for 14G CNB and 109 for 10G VAB. A comparison of surgical outcome and pre-operative biopsy outcome was used to determine diagnostic accuracy.

**Results** Statistical analysis found no significant relationship between CNB type and diagnostic accuracy (P = 0.211).

**Conclusion** There is no significant relationship between CNB type and diagnostic accuracy. Accurate targeting and representative MCC in specimen are key to optimal needle assessment of MCCs. 

**Implications for practice** The choice of CNB type for sampling MCCs should be based on the local unit biopsy performance, risk of complication and resource consideration especially with the current national shortages of BARD VAB and VAE needles.

### A study comparing Hologic LOCalizer seeds to wire guided localisation for clinically impalpable breast lesions

#### Sonali Shah, Natalie Allen, Vishnu Parameshwaran, Tamara Suaris

##### Barts Health NHS Trust, London, United Kingdom

###### Correspondence: Sonali Shah

*Breast Cancer Research* 23(2).

**Background** The mainstay of localisation of impalpable breast cancers prior to surgical excision is wire- guided localisation. The aim of our study is to evaluate the effectiveness of Hologic LOCalizer compared to Hawkin and Reidy wires at our institution in patients undergoing localised wide local exicision surgery (WLE) between 15/06/2019- 17/06/2021.

**Methods** **& Results** A statistical analysis was made between Hologic LOCalizer (n = 51) vs Reidy wires (n = 56) and vs Hawkins wires (n = 59) using Chi square and a two sample T-test. There is no significant difference in satisfactory (< 10 mm from lesion) localiser placement using Hologic LOCalizer vs Hawkins (p < 0.19) and Reidy (p < 0.74). The Hologic LOCalizer have no significant difference in positive margins rate vs Hawkins (p < 0.62) and Reidy (p < 0.8). Hologics LOCalizers are not significantly more likely to have operative difficulty excising the localiser vs Hawkins (p < 0.28) and Reidy (p < 0.3). WLE using Hologics LOCalizers are not significantly more likely to have unsuccessful excision of lesions vs Hawkins (p < 0.38) and Reidy. Excisions using Hologic LOCalizer do not have a significantly higher volume vs Hawkins (p < 0.18) and Reidy (p < 0.67), Excisions using Hologic LOCalizer do not have a significantly higher volume of excision:lesion ratio vs Hawkins(p < 0.21) and Reidy (p < 0.86). Smaller excision volume/lesion ratios are significantly associated with positive margins for excisions using Hologic (p < 0.03) and Hawkins (p < 0.04) but not Reidy (p < 0.54).

**Conclusion** Hologic LOCalizer WLE is non inferior to traditional wire localised WLE. During the covid-19 pandemic the increased use Hologic LOCalizer in our institute has allowed flexibility and improved surgical list utilisation

### Use of LOCalizer RFID tags in the pre-operative localisation of axillary lymph nodes

#### Sarrah El-Tahir^1^, Sharon Koo^1^, Robert Milligan^1^, Sunil Amonkar^1^, Alice Leaver^1^, Simon Lowes^1,2^

##### ^1^Gateshead Health NHS Foundation Trust, Gateshead, United Kingdom. ^2^Newcastle University, Newcastle upon Tyne, United Kingdom

###### Correspondence: Sarrah El-Tahir

*Breast Cancer Research* 23(2).

Our unit switched from using guidewire localisation of breast lesions to RFID tags in June 2019. We soon introduced RFID tags to localise metastatic nodes for targeted axillary dissection, which is becoming increasingly commonplace to excise clinically-relevant disease and minimise morbidity.

To assess the safety and efficacy of using RFID tags in the axilla, we reviewed our practice over a two-year period (June 2019-June 2021). In total, 679 RFID tags were deployed in 578 women. Of these, 31 tags were inserted into axillary nodes in 31 women aged 28–88, (mean 61); 9 targeted to clipped metastatic nodes post-NAC, 19 targeted to metastatic nodes in women receiving no other treatment prior to surgery, and 2 targeted to presumed non-malignant nodes (1 previous failed SNB and 1 persistent abnormal node on imaging with benign cores).

All tags and their respective nodes were successfully excised, confirmed by tag-site reaction. No complications were reported regarding tag insertion or retrieval.

An average of 4 nodes were excised per case (range 1–17, mode 2). In all expected cases, metastatic nodes were retrieved (all macrometastases). In 7 cases, there were no metastases; 5 were post-NAC (complete response confirmed in node) and 2 were expected benign nodes only (1 failed SNB and 1 persistent abnormal node proved reactive).

Six women had completion clearances for 1/1 (n = 1), 2/2 (n = 2), or > 2 nodes positive (n = 3), which produced only 2 further micrometastases in two women.

In conclusion, RFID tags are safe and effective to localise lymph nodes for targeted axillary dissection.

### Stereotactic insertion of LOCalizer RFID tags for pre-operative localisation of breast cancer

#### Sharon Koo^1^, Sarrah El Tahir^1^, Robert Milligan^1^, Sunil Amonkar^1^, Alice Leaver^1^, Simon Lowes^1,2^

##### ^1^Gateshead Health NHS Foundation Trust, Gateshead, United Kingdom. ^2^Newcastle University, Newcastle upon Tyne, United Kingdom

###### Correspondence: Sharon Koo

*Breast Cancer Research* 23(2).

Between June 2019-June 2021, since switching from guidewires to RFID tags, we have inserted 679 tags into 578 women. Where possible, we aim to insert tags under ultrasound guidance, even if the patient underwent initial diagnostic stereotactic VAB; occasionally, however, stereotactic tag insertion is necessitated. We reviewed our stereotactic insertions over this two-year period to evaluate its success.

Of 578 women, 67 had an initial diagnostic stereotactic biopsy. Of these, 47 had a successful ultrasound-guided RFID insertion targeting the VAB cavity/clip, and 20 underwent stereotactic RFID insertion. In these 20 women, 28 stereotactic tags were inserted: 13 women had a single tag to a single target, 5 women had 2 tags bracketing a single field of microcalcification, 1 woman had 3 tags bracketing microcalcification, and 1 woman had 2 tags targeting 2 separate lesions. An additional patient who initially underwent ultrasound-guided biopsy of her cancer also had stereotactic RFID insertion, where two mass lesions in the same breast were each targeted with a tag following complete radiological response to NAC as the gel clip was no longer visible on US/MRI.

In two cases, stereotactic tags were suboptimally positioned at the first attempt; one had a second tag inserted, effectively bracketing a large field of microcalcification, and the other had a guidewire on the morning of surgery. Surgery was deemed complete in all 21 patients at initial excision.

In conclusion, our initial experience indicates that stereotactic RFID tag insertion allows for effective targeting of lesions facilitating successful surgical excision.

## Scientific—Screening

### Audit of whole breast verses quadrant ultrasound in screening breast imaging

#### Lucinda Frank, Helen Massey

##### North Bristol NHS Trust, Bristol, United Kingdom

###### Correspondence: Lucinda Frank

*Breast Cancer Research* 23(2).

**Introduction** Currently our unit performs whole breast ultrasound (WBUS) at screening assessment clinic. Prior to changing practice to quadrant ultrasound in line with the 2019 RCR guidance on screening and symptomatic breast imaging we undertook an audit to evaluate whether WBUS identified any significant abnormalities not seen on mammogram/tomosynthesis.

**Method** The screening assessment notes of all patients diagnosed with a screen detected invasive cancer from April 2019 – March 2020 were reviewed. The following data was collected; whether the recalled abnormality was single or multiple, the findings on tomosynthesis and WBUS, the number of patients diagnosed with multi-focal malignancy and whether there were any significant lesions seen on WBUS that weren’t demonstrated on other modalities.

**Results** 250 cases met the inclusion criteria. 214 patients were recalled for a single abnormality on screening mammogram and 36 patients were recalled for complex/multiple lesions. 17 patients were diagnosed with multi-focal cancer. In 212/250 cases WBUS did not identify any further lesions. Of the 38 additional lesions seen on WBUS, 18 were demonstrated on mammogram/tomosynthesis and a further 18 were benign. Four patients were subjected to unnecessary biopsies following WBUS. There were no cases where WBUS significantly altered management.

**Conclusion** WBUS resulted in the diagnosis of 18 additional benign lesions and 4 unnecessary core biopsies. There were no cases where WBUS significantly changed patient management. We therefore recommend that it is safe for our unit to change to quadrant only ultrasound during screening assessment clinic in line with the RCR guidelines.

### A pilot analysis to determine impact of round length on interval cancer rates in the English Breast Screening Programme

#### Matthew Wallis^1^, Alison Murphy^2^, Kim Mellor^2^, Jackie Walton^2^, Mike Press^2^, Daniel Vulkan^3^, Stephen Duffy^3^, Olive Kearins^2^

##### ^1^Cambridge Breast Unit, Cambridge, United Kingdom. ^2^Screening QA Service PHE Screening, Birmingham, United Kingdom. ^3^Quenn Mary University, London, United Kingdom

###### Correspondence: Matthew Wallis

*Breast Cancer Research* 23(2).

**Introduction** English Breast Screening interval cancer data (for women screened between 2010 and 2016) is now published on Breast Screening Information System (BSIS). As in previous publications the rate between 24 and < 36 months is paradoxically only slightly higher than that of 12 to < 24 months. When plotted by month, the rate plateaus between 24 and 28 months and falls after 33 months. It has been presumed that this is a function of round length (actual time between screen and re screen/invitation). In preparation for more detailed analysis, we piloted a correction for round length to address this hypothesis.

**Method** Interval cancer data for women screened 2008–09 were obtained from Screening Histories Information Manager. Months since last invitation for the women invited for screening in January through March of 2011, 2012 and 2013 were obtained from breast screening repository data and used to correct the population denominator to recalculate interval cancer rates.

**Results** 2.1% of women were being invited within 12 months of their previous invitation, 5.5% by 24 months, 13.0% by 30 months and 97.4% by 36 months. Crude interval cancer rates were 0.56 per 1000 women screened at 0- < 12 months, 1.20 for 12- < 24 months and 1.37 for 24- < 36 months. After correction for ‘round length’, the rates were 0.56, 1.24 and 1.73 per 1000 respectively and no plateau was seen.

**Conclusion** Round length has a measurable impact on interval cancer rates. This need to be accounted for or corrected for in comparisons or analyses of interval cancer rates.

### “Hindsight is a wonderful thing”: A review of Interval Cancers diagnosed in our breast screening unit over a 14 month period

#### Matthew Murphy, Paula Hynam, Sarah Perrin, Emma Jackson

##### Musgrove Park Hospital, Taunton, United Kingdom

###### Correspondence: Matthew Murphy

*Breast Cancer Research* 23(2).

Interval cancers are tumours diagnosed between screening episodes within NHS breast screening programme (NHSBSP). The NHS BSP detects around 18,000 cancers annually, and approximately 6000 women present with interval cancers each year in England. Of these, approximately 80% have no abnormality on the previous screening mammograms, whilst 20% have detectable changes at screening1. These cases provide an excellent opportunity for learning, as the retrospectively evident mammographic abnormalities are often subtle.

From May 2020 to June 2021 a total of 78 interval cancers were identified in our unit. Of these, 54 (69%) were classified as category 1 (normal or benign mammographic features with no reason to recall the patient for assessment), and 24 cases (31%) were classified as category 2 (subtle mammographic signs present with hindsight). No category 3 cancers (obviously malignant mammographic appearances) were identified.

In this poster we provide an overview of the interval cancer classification. A breakdown of the subtle mammographic abnormalities evident within the category 2 cancers identified in our unit is provided, with examples shown, and trends and key learning points are discussed.

- Breast screening: reporting, classification and monitoring of interval cancers and cancers following previous assessment (Public Health England, 2021)

### Is It Practical to Vary Breast Screening Intervals According to the Risks of Developing Breast Cancer?

#### Wendy Tan, Susan Astley, Elaine Harkness, Gareth Evans

##### University of Manchester, Manchester, United Kingdom

###### Correspondence: Wendy Tan

*Breast Cancer Research* 23(2).

The NHS breast screening programme offers 3-yearly mammography for women aged 50–70. However, screening intervals are not stratified according to risk of developing cancer. This project aims to investigate the practical implications of risk-based screening. Data was collected from the PROCAS (Predicting Risk of breast Cancer at Screening) study and NHS Tameside breast screening round plan. Risk of breast cancer was calculated using the Tyrer-Cuzick model including factors such as hormonal replacement therapy, BMI and family history. It was assumed high-risk women would be screened annually, moderate-risk women biannually, whereas average and low-risk women would be screened triennially. Van locations and GP practices were plotted on a map in Tameside. Distance and time taken for women to travel to different van locations according to proposed screening intervals were calculated. The majority of women were average (70.9%) and low risk (19.3%), with only 8.6% at moderate and 1.2% at high risk. We found that using the stratified approach, women at high or moderate risk would have to travel a median of 2.64 additional miles (IQR 2.0,3.3) compared to traditional 3-yearly screening. Time to travel by car and bus respectively also increased by a median of 7.7 (IQR 6.0,9.0) and 18.5 min (IQR 8.6,23.9). The organisation of mobile screening units and additional travel time might present a challenge to a personalised breast screening approach. However, this only applies to 10% of women. Future studies should consider this when implementing a personalised approach to breast screening.

### Analysis of clinical, radiological, pathological features and outcome of mucinous breast carcinoma- retrospective study in a large UK teaching hospital

#### Amil Chaudhry, Pragya Singh, Jonathan Shadwell, Shams Vaquas, Abeer Shaaban, Shekhar Kaneri

##### Queen Elizabeth Hospital, Birmingham, United Kingdom

###### Correspondence: Pragya Singh

*Breast Cancer Research* 23(2).

**Background** Mucinous carcinoma represents about 4% of all invasive breast cancers and is more common in peri/postmenopausal women. Presentation is often symptomatic as palpable, well-defined masses and prognosis better than more common invasive cancers.

**Methods** Retrospective review of all cases of mucinous breast cancer treated at the Queen Elizabeth hospital, Birmingham in a 9 year period (March 2011 – Sept 2019) and analysis of relevant clinical, imaging, pathological features and the surgical treatment.

**Results**:

- **Clinical**: A total of 53 cases were reviewed (median age 70 years, range 38 – 93), including one male patient. 28/53 (53%) were screening patients, 18/53 (34%) diagnosed via symptomatic one-stop breast clinic and 7/53 (13%) as an incidental finding.
- **Radiological**: 48/49 (98%) of mammograms were abnormal. Mass/masses (average size 25.7 mm, range 5-97 mm) on 42/49 (86%) and asymmetric density on 6/49 (12%). On 45/47 breast ultrasounds (96%), a mass was identified. 8/50 (16%) showed metastatic axillary lymph nodes.
- **Pathological**: Histological grades and receptors were as follows: (grade I (20%), grade II (49%) and grade III (2%)) and receptor status [(98% (50/51) ER positive, 97.6% (41/42) PR positive and only 2% (1/51) HER2 positive].
- **Treatment**: 44/52 (85%) patients underwent surgical resection with majority (70%) undergoing wide local excision and 30% mastectomy. 6 /8 (75%) patients with metastatic axillary lymph nodes at presentation died by the time of data collection.

**Conclusion** Our data highlights a significant proportion of mucinous carcinomas diagnosed through the NHS breast screening programme and that nodal involvement at presentation is associated with poor outcome.

### The impact of the COVID-19 pandemic on the diagnosis of breast cancer in a symptomatic breast unit.

#### Paul Tansey^1^, Bryan Dalton^2^, Ronan McDermott^1^, Susannah Harte^1^, Sylvia O'Keeffe^1^, Mark Knox^1^

##### ^1^Dept. of Radiology, St James's Hospital, Dublin, Ireland. ^2^Centre for Advanced Medical Imaging (CAMI), Dublin, Ireland

###### Correspondence: Paul Tansey

*Breast Cancer Research* 23(2).

**Purpose** To evaluate the impact of the COVID-19 pandemic on the diagnosis of breast cancer in a symptomatic breast unit.

**Materials/Methods** We retrospectively collected data on symptomatic patients newly diagnosed from March 2019–2020 (pre-COVID/Group-1) and March 2020–2021(COVID/Group-2). We analysed age at diagnosis and UICC radiological staging (TNM).

**Results** There was no significant change in the number of patients in each study group (n = 268 vs n = 254, p > 0.05), or the age distribution of patients diagnosed with breast cancer after the onset of the pandemic. Prior to the pandemic, 32.5% of patients (n = 87) were under 50 compared to 39.4% (n = 100) after, with a corresponding decrease in women 50 years or over, 67.5% (n = 181) compared to 60.6% (n = 154) after the onset. There was a statistically significant increase in the proportion of women diagnosed with Stage IIIA disease and higher in the < 50 age group after the onset of the pandemic when compared to before, (n = 10(3.7%) vs n = 28(11%) (p < 0.05)). There was a non-significant increase in stage IV cancers in the > 50 cohort after the onset of the pandemic (p > 0.05).

**Conclusion** While there was no significant change in the age distribution of patients diagnosed with breast cancer in our hospital after the onset of the pandemic, there was a significant increase in the proportion of women in the < 50 age group presenting with Stage IIIA disease and above. Further investigation is required to observe the impact of the pandemic on the presentation of women with breast cancer to breast cancer services.

### Analysis of B5a upgrades in a large screening centre - our experience over 3 years

#### Hwee Fang Lim^1^, Gauri Babu^2^

##### ^1^NHS Lothian, Edinburgh, United Kingdom. ^2^NHS LOTHIAN, Edinburgh, United Kingdom

###### Correspondence: Hwee Fang Lim

*Breast Cancer Research* 23(2).

Ductal carcinoma in situ (DCIS) accounts for as many as 30% of breast cancers detected in screening populations. 20% of the women who had DCIS diagnosed on preoperative breast biopsies, classified as B5a (non-invasive), were subsequently found to have invasive cancer at surgery.

We reviewed our experience of B5a upgrades in one screening cycle and did a comparative analysis with previous data from our centre. The results were also compared to a national audit. A total of 28 cases of B5a upgrade within this period were identified from our centre.

Our findings demonstrated the main type of mammographic abnormality for B5a upgrades is microcalcifications. The biopsies were predominantly performed using large volume vacuum assisted device. The majority of the B5a upgrades usually involve small invasive tumors found within large areas of DCIS. Among this High grade DCIS was the commonest type at final surgery. We also noted that only 59% of the patients with microcalcifications as the primary mammographic abnormality proceeded to have a subsequent ultrasound scan. A normal result on the scan was frequently noted in a majority. This audit raised a discussion if we should consider performing ultrasound in extensive areas of calcifications or certain morphological types of calcification..

Review of literature shows there is no standardization of practice or consensus on this subject. We will re-audit our experience in the next screening cycle.

## Scientific—Staging/Systemic Imaging

### Audit: Indications for staging CT in breast cancer as per ESMO and RCR guidelines

#### Michelle Siu, Reena Aggarwal, Manal Al-Kaiem, Miaad Al-Attar.

##### University Hospitals of Leicester, Leicester, United Kingdom

###### Correspondence: Michelle Siu

*Breast Cancer Research* 23(2).

In our institution, primary staging CT is performed in all breast cancer patients undergoing neoadjuvant/adjuvant chemotherapy irrespective of tumour stage/biology and nodal status. We audited our practice against ESMO and RCR guidelines which only indicate a CT in ≥ T3 tumours, node positive (≥ 4 nodes in RCR, any number in ESMO), aggressive biology (ESMO only) and clinical evidence of metastases. This aligns with recent literature that recommends staging CT can be omitted in < T3 and node negative cancers. Performing unnecessary CTs increases costs and can lead to false positive results, increasing patient anxiety and prolonging the patient pathway. Target 100% compliance.

129 CTs were identified over 17 months retrospectively and prospectively using MDT minutes and CRIS. Data was collected on patient demographics, tumour status and CT findings. 12% were positive for metastases. For ESMO guidelines: 93% were compliant and all 9 of the non-compliant scans were negative for metastases (1 showed an incidental pancreatic primary). For RCR guidelines: 35% were compliant and 1/40 of the non-compliant scans was positive. Overall 19% demonstrated false positive findings with 35 follow-up investigations generated.

In conclusion, compliance with ESMO guidelines was reasonable but poor with RCR guidelines. There were no positive scans in tumours < T3 and node negative which improves confidence that these guidelines will not reduce sensitivity. If the RCR guidelines were followed, one positive scan would be missed. The results were presented at the multidisciplinary departmental protocol meeting and the ESMO guidelines adopted. A re-audit will be completed in a year.

### Uncommon sites of breast cancer metastasis—A pictorial review

#### Juilee Prabhu, Nameet Hattangadi

##### Barking Havering Redbridge University Hospitals, Romford, United Kingdom

###### Correspondence: Juilee Prabhu

*Breast Cancer Research* 23(2).

Breast cancer recurrences are of three types- local, regional and distant. Distant metastasis occur several years or so after breast cancer, although sometimes are diagnosed at the same time. Invasive ductal carcinoma is the most common type of cancer(85%) and most common sites of distant metastasis being lung, liver, bone and brain. Early detection and treatment improve prognosis. Here we describe metastasis of breast cancer to uncommon sites, rate of which is on rise due to more effective therapy prolonging survival and early detection on imaging.

These cases were discussed in the metastatic MDT. We present cases of metastasis of breast cancer to Orbit, Stomach, Salivary Gland, Ovary and Urinary Bladder. The propensity of metastasis at these sites occur in frequency of < 1%.

Evaluation of patient reported symptoms is essential in detecting these as early as possible to improve survival. Hence knowledge of even rare sites of breast cancer metastasis is of paramount importance for clinical interpretation of new symptoms in breast cancer survivors.

## Scientific—Symptomatic

### Should we routinely perform biopsy in patients with suspicion of fat necrosis?- biopsy results versus US findings in patients attending one stop clinic at UHNM.

#### Amira Helal, Zatinahhayu Mohd-Isa, Aleksandra Stankiewicz.

##### University Hospital of North Midlands, Stoke-on-Trent, United Kingdom.

###### Correspondence: Amira Helal

*Breast Cancer Research* 23(2).

Fat necrosis is a well-described benign entity presenting as a non-suppurative inflammatory process of adipose breast tissue, which may related to previous trauma, biopsy, radiotherapy, surgery, breast reconstruction or fat grafting. Clinically patients can be asymptomatic or presenting with a single lump, multiple smooth round nodules, or irregular masses with skin retraction.

Imaging appearances of fat necrosis depend on its stage of evolution, from hyperechoic masses initially, through cystic degeneration commonly known as oil-cysts, to calcifications and fibrosis occurring months or years after initial presentation.

The diagnosis can be confirmed on the basis of either serial imaging studies showing chronological changes compatible with the evolution of fat necrosis or improvement in clinical symptoms.

The aim of this study to assess usefulness of to biopsy or follow up lesions with typical US of fat necrosis /bruise/haematoma) in cases of positive history of trauma or operation.

283 patients presented through 2ww or assessment clinic for ? fat necrosis. Out of those, 25 cases were referred for follow up, aspiration of oil cysts was performed in 13 and biopsy in 33 cases. Histopathological and radiological results were compared.

In 2 patients with history of breast cancer surgery, biopsy showed malignancy. For suspected post traumatic fat necrosis, all biopsy findings were benign.

Therefore, in post traumatic cases with typical US features of fat necrosis no further investigation is required. However, care should be taken in postoperative cases with history of malignancy as recurrence and fat necrosis could have similar appearance.

### Breast cancer mimics- the radiological pitfalls in commonly encountered cutaneous lesions

#### Elliot Elwood^1^, Julie Scudder^2^, Victoria Sinnett^2^, Romney Pope^3^

##### ^1^Chelsea and Westminster Hospital, London, United Kingdom. ^2^The Royal Marsden Hospital, London, United Kingdom. ^3^Royal Marsden Hospital, London, United Kingdom

###### Correspondence: Elliot Elwood

*Breast Cancer Research* 23(2).

Palpable and/or visible cutaneous lesions are a common presentation to the symptomatic breast service and can easily be mentally dismissed as benign even before assessment. Although the majority of skin nodules represent benign lesions; most commonly sebaceous cysts, the possibility of a malignant lesions should not be readily dismissed. We present four such cases; initially assessed and determined benign which subsequently proved malignant. We review the discriminating imaging features of benign sebaceous cysts and cutaneous breast malignancy and highlight the falsely reassuring features. There is significant overlap in imaging features throughout he presented cases, highlighting the need for caution when assessing seemingly benign cutaneous breast lesions.

## Service Improvement

### Demonstration of the positive Impact of Digital Breast Tomosynthesis (DBT) inclusion into a large regional Breast Screening Assessment Service demonstrating reduced overall and benign biopsy rates whilst maintaining cancer diagnosis in women recalled from the National Breast Screening Service (NBSS).

#### Miaad Al-Attar^1^, Lisa Grosvenor^2^, Monika Stovickova^1^, Nicola Hartley^1^, Gayle McDonald^1^, Moin Hoosein^1^, Lakshmi Sundaram^1^, Diane Lister^1^

##### ^1^Breast Care Centre, Glenfield Hospital, UHL, Leicester, United Kingdom. ^2^Breast Care Centre, Glenfield Hospital, UHL, Leicester, United Kingdom

###### Correspondence: Miaad Al-Attar

*Breast Cancer Research* 23(2).

Digital Breast Tomosynthesis (DBT) is a well-established modality within UK screening units for assessment of recalled women.

We reviewed the impact on our practice of reduction in benign biopsy rates and discharge without biopsy, following introduction of DBT to our large screening unit (population 177,000 per screening round) by retrospective reviewing complete annual assessment data 2018.

Multiple data points were recorded, including morphology, size, biopsy and overall assessment outcome.

Within the review period, 1126 women recalled were assessed with DBT. Within this cohort, 1191 areas of interest had been identified on full field digital mammography (FFDM). 56% (672) of the FFDM areas of concern were not identified at DBT and were discharged with no needle intervention, following completion of assessment process. This compares to no biopsy in 48% of recalled areas in the year prior to DBT implementation, equating to an approximately reduction of 100 biopsies.

Over 70% of cases, discharged from assessment following DBT with no intervention were classified on FFDM as well defined masses (WDM) or asymmetric densities (ASD). Of lesions classified as WDM/ASD and biopsied following DBT, there was a low malignancy yield (< 5%). Of WDM/ASD biopsied, 90% had a benign histological outcome.

As in previous studies, DBT accurately identified true parenchymal deformities (PD). Areas of FFDM concern confirmed on DBT as spiculate, ill-defined or PD were associated with highest incidence of malignancy following DBT assessment (> 90%).

DBT in our unit has been shown to accurately characterise lesions, reducing needle intervention whilst maintaining cancer diagnosis.

### SAVISCOUT radar reflector: a new and advanced method of lesion localisation. Our initial experience in breast and axilla

#### Shaista Siddiqui, Sheetal Sharma, Asha Shivaram, Julia Henderson.

##### Royal Liverpool University Hospital, Liverpool, United Kingdom.

###### Correspondence: Shaista Siddiqui

*Breast Cancer Research* 23(2).

Breast lesion localisation is an integral part of breast radiology, enabling surgeons to accurately remove the abnormal tissue during surgery. Our previous practice, using wire insertions or ROLL (radio-guided occult lesion localisation) required localisations to be performed on the morning of surgery, which was restrictive and often caused disruption to morning clinics. We were therefore keen to find a method of localisation allowing us to uncouple it from the day of surgery.

Saviscout is a radiofrequency reflector for impalpable breast and axillary lesions. It can be inserted under ultrasound or stereotactic guidance and can be placed months in advance of surgery, providing particular benefit in patients undergoing neoadjuvant treatment. It is inserted using a pre-loaded 16G needle. After insertion, Saviscout is detected in situ using a Saviscout probe and console to confirm deployment. It is detected peri-operatively using a similar probe and console.

In this poster we will describe our experience of Saviscout localisation since 2020, in both breast and axilla. The data of our first 100 patients is promising and we are in the process of analysing data from around 250 patients. So far, we have had no failures and very few post-surgical margin-positive cases. This method has proved equally successful for axillary lymph node localisation.

Our experience to date is that Saviscout has a clear advantage over wire placements and ROLL without compromising accuracy.

We will present data to show that Saviscout is an easy-to-use method of localisation which is accurate and convenient for both radiologists and surgeons.

### Breast lesions incidentally detected by Cross Sectional Imaging: Impact and effectiveness of our Local Management Algorithm

#### Jennifer Royds, Gauripriya Babu, Vicky Tilliridou

##### NHS Lothian: Mammography X-ray, Edinburgh Breast Unit, Western General Hospital, Edinburgh, United Kingdom

###### Correspondence: Jennifer Royds

*Breast Cancer Research* 23(2).

**Aim** In 2016, we presented our local experience of incidentally detected breast lesions, and our proposed management algorithm. Our algorithm pilot launched in May 2020, during the peak of the COVID19 pandemic. Our study evaluates the impact and effectiveness of the algorithm, and suggests potential areas for development.

**Methods** Imaging and pathological outcomes of patients referred into the pathway by either reporting radiologists or breast clinicians were analysed. We also evaluated the triage accuracy and timeframes.

**Results** 76 cases were analysed. 8 (10%) required cyst aspiration, 28 (37%) were normal or declined investigation (3), 40 (53%) required biopsy. Biopsy results comprised: 10% B1(hamartoma, normal asymmetry), 33% B2 (reactive nodes, fibroadenoma, fat necrosis) 7% B3 (papillomas), 50% malignant (17 invasive breast cancer, 3 metastatic nodes from breast, lung or skin cancer). Average interval between provoking CT and breast imaging was 27 days, between pathway referral and breast imaging was 13 days. Timeframes were well within the 4–6 week local wait for new patient clinic assessment and the Scottish Government 61 day diagnostic target. Patients triaged by breast radiologists as malignant or indeterminate / suspicious had shorter waits than those triaged benign. No malignant lesions were triaged benign, all were appropriately triaged as indeterminate or above.

**Conclusion** Our local pathway for incidental breast lesions is fast and effective. During pandemic times it has allowed safe planning of appropriately triaged work, out with the new patient clinic. Further promotion and development will ease new patient pressure and allow faster, accurate assessment of patients.

### Identification of incidental breast cancers by radiologists

#### Claire Filippini, Louise Wilkinson

##### Oxford University Hospitals NHS Foundation Trust, Oxford, United Kingdom

###### Correspondence: Claire Filippini

*Breast Cancer Research* 23(2).

Early diagnosis of breast cancer (BC) leads to better treatment options. As radiologists, we are in a unique position to spot undiagnosed tumours on CT scans performed for other reasons e.g. CTPA. This audit was performed on a random sample of 892 women, diagnosed with BC between 2011–2021, aged > 70 years at the time of diagnosis. Of these women, we looked to see who had had a CT scan prior to their BC diagnosis which demonstrated a breast tumour that could have been identified by the reporting radiologist.

123 CT scans were performed in these 892 women prior to their diagnosis of BC in which there was an opportunity to identify a breast mass. 49% of visible tumours were reported by the radiologist. However, 37% of visible tumours were not reported at all by the radiologist, whilst another 14% of tumours were not reported at the earliest opportunity, but were eventually spotted.

Therefore, in 51% of cases where a tumour could have been identified, it was either not reported at all, or not reported at the first available opportunity.

This highlights the importance of a review of the breasts on all cross sectional imaging performed, whatever the indication, in order to identify incidental BC and to improve the outcomes of treatment which can be initiated at the earliest opportunity.

### High risk screening breast MRI: is adequate sensitivity possible with acceptable recall rates?

#### Lucinda Frank, Carina Brolund-Napier, Richard Sidebottom, Iain Lyburn, Sarah Vinnicombe.

##### Thirlestaine Breast Centre, Cheltenham, United Kingdom.

###### Correspondence: Sarah Vinnicombe

*Breast Cancer Research* 23(2).

**Introduction** Women with genetic mutations, notably BRCA mutations, and those who have had previous thoracic radiotherapy at a young age are at very high risk of developing breast cancer and are entitled to annual screening with MRI ± mammography. Further assessment is recommended for MRI detected indeterminate or suspicious masses ≥ 5 mm, or non-mass enhancement ≥ 10 mm. The minimum standard for recall rate is < 10% with an expected standard of < 7%. Nationally, many units have struggled to achieve this. This audit aimed to review recall rates and outcomes in a single breast screening unit.

**Method** All high risk screening MRIs conducted between January 2014 and September 2019 were reviewed including classification, type of follow up imaging and any biopsy results.

**Results** There were 283 screening episodes between January 2014 and September 2019. Nineteen patients were recalled (12 prevalent screens, 7 incident screens), for an overall recall rate of 6.7%. The recall rate per year varied from 3 to 9% with no discernible trend. Five cancers were diagnosed (cancer detection rate 17.6/1,000; PPV1 26%). On retrospective review of recalled cases, 3 were deemed unnecessary. All 3 were before a protocol change that included high resolution T2 weighted sequences and diffusion weighted imaging. In one case, DWI was misinterpreted.

**Conclusion** In a centre with a small number of experienced MRI reporters and a rigorous protocol, it is possible to meet the expected recall rate standard. Diffusion weighted imaging is invaluable for increased specificity, especially in prevalent screens.

